# The Short Term Musculoskeletal and Cognitive Effects of Prolonged Sitting During Office Computer Work

**DOI:** 10.3390/ijerph15081678

**Published:** 2018-08-07

**Authors:** Richelle Baker, Pieter Coenen, Erin Howie, Ann Williamson, Leon Straker

**Affiliations:** 1 School of Physiotherapy and Exercise Science, Faculty of Health Science, Curtin University, Perth 6102, Australia; richelle@rabc.com.au (R.B.); p.coenen@vumc.nl (P.C.); ekhowie@uark.edu (E.H.); 2 Department of Public and Occupational Health, Amsterdam Public Health Research Institute, VU University Medical Center, 1081 Amsterdam, The Netherlands; 3 Department of Health, Human Performance and Recreation, University of Arkansas, Fayetteville, AR 72701, USA; 4 School of Aviation, Faculty of Science, University of New South Wales, Sydney 2052, Australia; a.williamson@unsw.edu.au

**Keywords:** human-computer interaction, musculoskeletal disorders, biomechanics, mental work capacity, office ergonomics

## Abstract

Office workers are exposed to high levels of sedentary time. In addition to cardio-vascular and metabolic health risks, this sedentary time may have musculoskeletal and/or cognitive impacts on office workers. Participants (n = 20) undertook two hours of laboratory-based sitting computer work to investigate changes in discomfort and cognitive function (sustained attention and problem solving), along with muscle fatigue, movement and mental state. Over time, discomfort increased in all body areas (total body IRR [95% confidence interval]: 1.43 [1.33–1.53]) reaching clinically meaningful levels in the low back and hip/thigh/buttock areas. Creative problem solving errors increased (β = 0.25 [0.03–1.47]) while sustained attention did not change. There was no change in erector spinae, trapezius, rectus femoris, biceps femoris and external oblique median frequency or amplitude; low back angle changed towards less lordosis, pelvis movement increased, and mental state deteriorated. There were no substantial correlations between discomfort and cognitive function. The observed changes suggest prolonged sitting may have consequences for musculoskeletal discomfort and cognitive function and breaks to interrupt prolonged sitting are recommended.

## 1. Introduction

A rapidly increasing body of evidence supports an association between sedentary behaviour and the risk of adverse health outcomes [1]. These include negative cardiometabolic outcomes such as type two diabetes [2], and some cancers [3]. In addition there is epidemiological evidence of increased risk of premature mortality [4,5] and obesity [6] however this is inconclusive. As sedentary (e.g., office) jobs become more prevalent [5] the health risks for office workers are an increasing concern for society and industry. However the impacts of prolonged sitting on musculoskeletal discomfort across the body and on cognitive function are not yet clear. 

Prolonged sitting is a potential hazard for workers’ musculoskeletal health [7,8]. For the low back there is mixed evidence regarding the association between sitting at work and low back pain [9,10]. Laboratory studies have found increased discomfort (if not pain) in the low back with prolonged sitting [11,12]. In understanding why discomfort may arise, one hypothesis suggests sustained low level activation and loading of passive tissues [13] to be responsible. Other hypotheses include postural changes such as flattening of the lumbar lordotic curve with increased sitting time [14] and chronic muscle deconditioning due to habitually lower levels of activation [13] leading to muscle fatigue with prolonged low loading in static postures. In order to understand why discomfort occurs, further research on muscle fatigue and postural factors possibly contributing to the development of discomfort is required.

Whilst a causal relationship between prolonged sitting and work-related musculoskeletal disorders of the lower limbs is not clear [15], a number of individual studies have found associations. Studies suggest there may be an association between sitting and buttock pressure and discomfort [16,17]. Laboratory studies of prolonged sitting have also reported lower limb discomfort [11] and suggested a link with lower limb swelling [18,19]. During prolonged sitting there is typically minimal leg muscle activity, compared to during more active work positions such as walking or cycling, which may impact vascular return [17] causing leg swelling. Further, there is a passive load on tissues particularly at the buttock but also the thigh [20]. A better understanding of these multifaceted mechanisms including muscle activity and leg swelling which could contribute to discomfort may assist with developing clear protocols to prevent or minimise lower limb discomfort where prolonged sitting is required. 

Neck and upper limb symptoms among office workers have been studied more widely, however evidence of an association is mixed. Wærsted et al. [21] concluded from a systematic review that there was limited epidemiological evidence for an association between computer work and neck disorders. For example, Gerr et al. [22] tracked 632 new computer users and found over 50% reported neck and upper limb musculoskeletal issues within 12 months. In considering just the upper limb, da Costa and Vieira [15] found reasonable evidence supporting computer work to be a risk for wrist/hand discomfort in a systematic review, however they reported a lack of conclusive evidence for shoulder and elbow discomfort. In a field study by Roelofs and Straker [23] bank tellers had increased discomfort in the upper limb with just-sitting for one day compared to other work positions while a two week study by Davis and Kotowski [24] found greater discomfort in the upper limb for call centre workers in sitting postures compared to sit-stand work postures. Laboratory studies have also found an increase in neck and shoulder discomfort associated with prolonged sitting [11,16]. Despite the lack of consensus of the risk office work presents for the upper limb, it is clear discomfort is evident for some office workers. This has been postulated to be due to increased demands on postural musculature due to the arm being unsupported over prolonged periods [23], as well as repetitive movement and increased muscle activity associated with computer work [21]. Further clarity of how factors influence upper limb discomfort during sitting will support guidance to industry.

In addition to musculoskeletal risks, concern has also been raised about the impact of sedentary behaviour on cognition, which has potential to affect office workers’ performance. Emerging evidence suggests there may be a negative association [25,26] between habitual sedentary behaviour and cognition. Considering acute effects, Hasegawa et al. [27] found longer task time during prolonged sitting (90 min) resulted in lower work performance. Mental state has also been considered in laboratory studies with self-reported fatigue levels being higher during prolonged sitting compared to other work positions [28,29]. Field studies which considered sitting compared to a sit-stand work position found sitting resulted in more fatigue and self-rated lower energy level [30] as well as reduced focus and productivity [31]. Evidence suggests that higher levels of physical activity, such as during exercise, can influence brain function in the short term through acute physiological response including increases in heart rate, oxygen uptake, respiration and blood flow including cerebral blood flow [32]. From a longer term perspective higher levels of habitual physical activity have been associated with better levels of cognitive function [26]. Thus, sitting (with a relatively low energy expenditure [33]) has potential to result in a decline of cognitive function over time. For knowledge based occupations (such as office workers) where prolonged sitting is required, an understanding of how cognitive function may change over time would assist in guiding recommendations to optimise work performance. 

An increasing evidence base suggests there may be health risks from prolonged sitting. Further there may be an increased risk of musculoskeletal discomfort and cognitive decrement. The current study aimed to examine discomfort and two areas of cognitive function over two hours of prolonged sitting. It was anticipated that discomfort would increase and cognitive function would decrease during this period. Sustained attention and more the complex cognitive function of problem solving were selected as cognitive functions likely to be important for knowledge based office work and there may have been differential effects on lower versus higher order cognitive function. Additional factors of muscle fatigue, low back angle, pelvis movement and mental state were also measured to explore potential mechanisms underlying these anticipated changes. As it was expected that discomfort may affect cognitive function the correlation between these variables was also explored.

## 2. Method

A convenience sample of twenty adults was recruited via personal and professional networks including through a university physiotherapy department. Male participants (n = 7) were aged 32 (SD 9.3, range: 20–45 years) years, with weight 79.6 (4.4) kg and height 180.6 (6.2) cm while female participants (n = 13, noting one participant chose not to provide age/weight/height data) were aged 36.2 (7.6, 20–45 years) years, with weight 64.2 (15.4) kg and height 166.5 (7.3) cm. All male participants self-identified as undertaking a sedentary occupation while for females 10 identified as sedentary, one as standing and one as undertaking physical work. The inclusion criteria were between 18–65 years of age, English and computer literacy and physical ability to undertake sitting for two hours. Exclusion criteria were those for whom workstation set-up was anthropometrically unsuited due to height or girth and those who had known pre-existing pain. One potential participant was excluded.

### 2.1. Design and Procedure

This laboratory-based study had a repeated measures design. Participants sat for two hours and were encouraged to remain sitting but were able to fidget or stand briefly if they needed to due to discomfort. Measurements were taken during participant’s usual sitting posture (without postural prompting).The independent variable was time sitting and dependent variables were discomfort, cognitive function (creative problem solving and sustained attention), muscle fatigue, low back angle, pelvis movement and mental state. Measures of all dependent variables were taken at commencement and repeated at 30 min intervals (five measures in total). Participants visited the laboratory prior to study commencement to be familiarised with the procedure and tests. 

**Figure 1:**
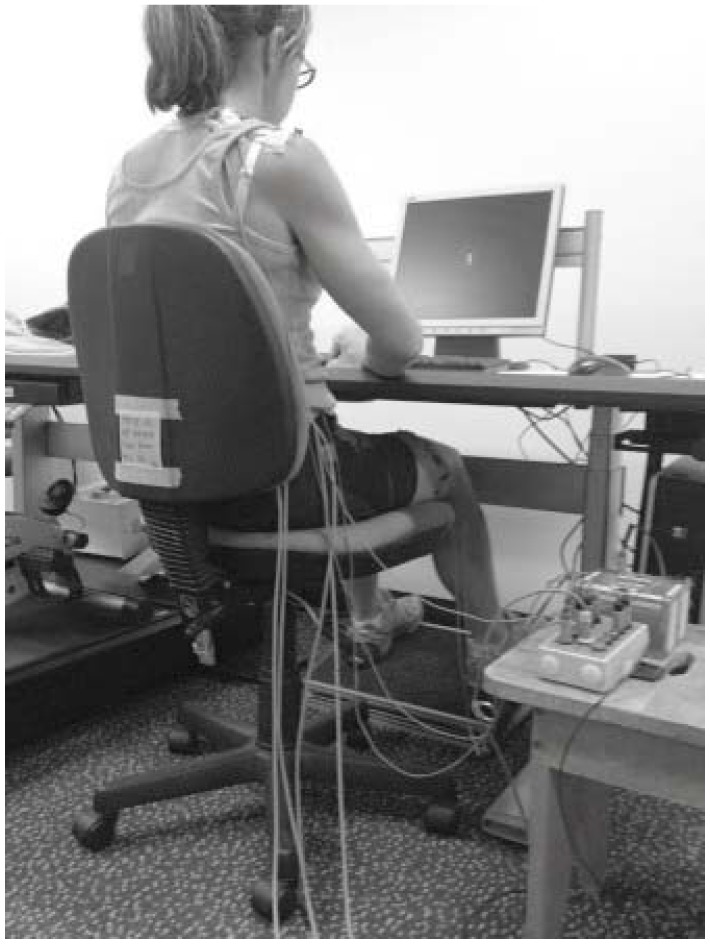
Work position of participants.

Participants undertook self-directed computer or paper based activity each two hour period. A desk (A7TR78928H, Steelcase, Sydney, Australia) was adjusted to allow 90° elbow flexion with fingers resting on the home row of the keyboard. The forearms were able to rest on the desk surface with a close to neutral wrist position. A standard adjustable office chair with backrest was used. The top of the computer screen (15 inch, Acer, Taiwan) was altered to participant eye level and a height adjustable footrest (Z rest, Ergolink, Perth, Australia) was used by all participants to allow 90° knee flexion (see Figure 1).

### 2.2. Dependent Variables

#### 2.2.1. Discomfort

Participants rated intensity of musculoskeletal discomfort using an electronic (modified) version of the Nordic Musculoskeletal Questionnaire (NMQ). Nine body areas were rated against anchors 0 = ‘*no discomfort*’ and 100 = ‘*discomfort as bad as it can be*’. The NMQ has been used extensively to identify location and intensity of musculoskeletal discomfort with acceptable reliability [34]. Combined scores were calculated (averaging body areas) for upper limb (shoulder, elbow, wrist/hand), lower limb (hip/thigh/buttock, knee, ankle/feet) and total body (all scores).

#### 2.2.2. Cognitive Function

The Ruff Figural Fluency Test (RFFT) was used to examine problem solving [35]. The RFFT was chosen as a test which was not overtly novel, thereby avoiding unduly altering attentional level as a result of the testing process [36]. Participants were required to join five dots, within a defined box, to create as many unique designs as possible for one minute per part. Each part had a maximum of 35 possible designs. Two consecutive parts of the five part RFFT were completed each testing session. Participants used their computer mouse to draw the designs, with total number of designs and errors (repeat of design or not within the rules) manually tallied by the researcher. The rules required a design to be contained to that box and not enter a neighbouring box or interlink with a neighbouring figure. Designs with alternate orientation (rotation) were considered unique. The RFFT has shown inter-rater reliability of scoring for unique designs of 0.98 (intra-class correlation coefficients) and for perseveration errors of 0.94 and has evidence of convergent validity with other executive function tests [35]. 

Sustained attention was measured using a Go/No-go test, the Sustained Attention to Response Test (SART) (http://www.millisecond.com/download/library/SART/). The SART has been widely used [37] and requires participants to press the spacebar for all the digits which flash briefly (250 ms) on the screen (Go response), except the number three (No-go response), over a period of 4 min 20 s. Participants were instructed to respond as quickly as possible whilst concurrently aiming to minimise errors. No-go success (%) and response time (millisecond) were used for analysis.

#### 2.2.3. Mental State

A scale based on the Visual Analogue Scale for Fatigue, which has evidence for reliability and validity [38], was used. The scale consisted of five visual analogue items with anchors of: ‘*not at all alert/tired/drowsy/fatigued’* to *‘extremely alert/tired/drowsy/fatigued*’ and ‘*concentrating was no effort at all*’ to ‘*concentrating was a tremendous chore*’. The scales were computer administered with participants using a mouse to mark their perception. Scores from all items were averaged and normalised to a 0–100 scale for further analysis as a measure of mental state.

#### 2.2.4. Muscle Fatigue

Muscle activity data was collected for 10 s using surface electromyography (EMG), via Octopus AMT-8 EMG Cable Telemetry System (Bortec Electronics Inc., Calgary, AB, Canada), with a sample rate of 2000 Hz. Skin preparation was undertaken (area shaved, cleaned with ethyl alcohol and lightly abraded with fine sand paper) before self-adhesive disposable Ag/AgCl (6 mm gel diameter) electrodes (Neuroplus, Vermed, New York, NY, USA) were secured with tape over the following muscles: right side upper trapezius (with 20 mm centre to centre distance 20 mm lateral to the midpoint between the acromion process and C7 spinous process [39]), external oblique (just below the rib cage and along a line connecting the most inferior point of the costal margin and the contralateral pubic tubercle [40]), lumbar erector spinae (iliocostalis lumborum pars thoracis at L1 spinous process level midway between the midline and the lateral aspect [41]), rectus femoris (midway along a line between the anterior superior iliac spine and superior border of the patella [42]) and biceps femoris (midway laterally on the posterior part of the thigh [42]). The common earth electrode was placed on the acromium. 

Muscle activity was normalised against submaximal reference voluntary contractions (held for three seconds, repeated three times for each muscle) as follows: upper trapezius (elevating the upper arm in 90° abduction in the scapular plane while seated [39]), external oblique (in supine with hips flexed to 45° and knees flexed to 90° performing a double leg raise 1 cm off the supporting surface [40]), erector spinae and biceps femoris (lying prone position with knees bent to 90° and both knees lifted 5 cm off the supporting surface [40]), rectus femoris (sitting with hips flexed to 90° and the tested knee extended to 45° [43] with 2 kg weight secured at ankle).

EMG data was band pass filtered (high 10 Hz and low 1000 Hz) by the amplifier. A customised program (LabView, National Instruments Inc., Austin, TX, USA) was then used to process the EMG data including demeaning, rectifying and finally visual inspection. Muscle fatigue was operationalised using median frequency and normalised amplitude. Amplitude and/or frequency measures have been widely used to indicate muscle fatigue while undertaking prolonged postures [44,45]. Mean median frequency and normalised mean amplitude (as a percentage of middle submaximum voluntary reference contraction) were calculated for each sample and used for further statistical analysis. Reliability and validity of these measures has previously been demonstrated in our laboratory [40]. Outliers (>1.5 times the interquartile range) were removed. 

#### 2.2.5. Low Back Angle and Pelvis Movement

Low back angle and pelvis movement were measured using 3 Space Fastrak (Polhemus Navigation Sciences Division, Vermont, VT, USA) with 10 s samples (at 25 Hz) (in line with Gallagher and Callaghan [46]. Fastrak is an electromagnetic device which generates a low frequency magnetic field and determines the position and orientation of sensors relative to the field source [47]. Sensors at T12, L1 and S2 (based on the protocol by Levine and Whittle [48]) were secured over spinous processes. The earlier mentioned Labview program calculated a total low back angle (as the angle between T12 and S2 in the sagittal plane) and pelvis movement (as the distance, in centimetres, of transverse plane displacement of the S2 sensor [41]) for analysis.

### 2.3. Statistical Analysis

Mixed-models with random intercepts for participants were used to assess changes over time (with five repeated measures over two hours as independent variable) for each of the dependent variables. Data were examined for normality via histogram, and kurtosis and skew statistics. For normally distributed data (cognitive function including problem solving and sustained attention, low back angle and perceived mental state) linear models were used. Skewed data (muscle fatigue, pelvis movement) were logarithmically transformed and then used in linear models (tables present back transformed data). Negative binomial models were used for data with a count distribution (discomfort). Betas (for linear models) and incident rate ratios (IRR, for negative binomial models) together with 95th percent confidence intervals and *p*-values are reported depicting the change in the respective dependent variables over time. Changes in discomfort greater than 10/100 were considered clinically meaningful based on Hägg et al. [49] and tested with pairwise comparisons to baseline discomfort using negative binomial models. 

To explore potential mechanisms, correlations were examined between changes (measures at baseline compared to 120 min) over the two hours period for low back discomfort (with erector spinae and external oblique amplitude and median frequency, low back angle in sagittal plane and pelvis movement in transverse plane), lower limb discomfort (with biceps femoris and rectus femoris amplitude and median frequency and pelvis movement) and upper limb discomfort (with trapezius amplitude and median frequency and pelvis movement). In addition, correlation of total body discomfort with the two areas of cognitive function and mental state were examined. Pearson (normally distributed data) and Spearman (non-normal data) tests were used to assess correlations.

In all analyses, statistical significance was accepted at alpha probability of *p* < 0.05. The software used for analysis was STATA (StataCorp 2015, Stata Statistical Software: Release 14. StataCorp LP, College Station, TX, USA). Correlations were categorised according to weak r < 0.29, moderate r = 0.30–0.49, and substantial r > 0.5 [50].

## 3. Results 

One participant elected to stand briefly once (after completing discomfort rating at the 60 min time point). At no time during the two hours did the discomfort ratings of this participant reach clinically meaningful levels in any body region (highest rating was 4/100).

**Figure 2:**
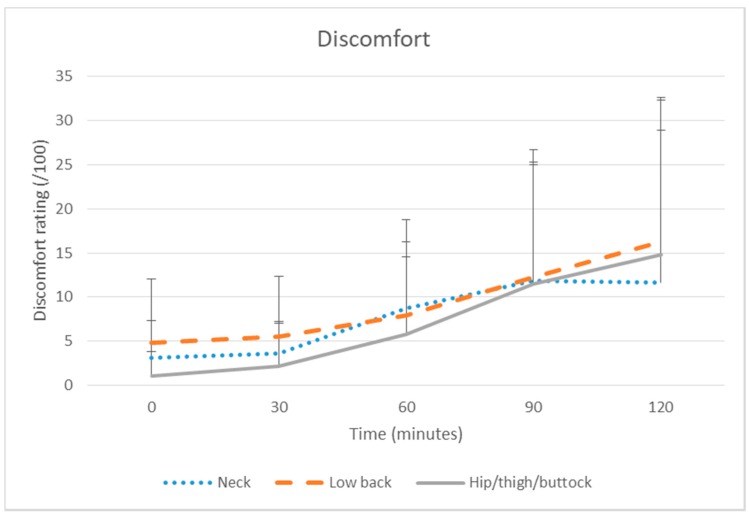
Discomfort (mean + standard error) for neck, low back and hip/thigh/buttock over two hours prolonged sitting (non transformed data).

Discomfort increased significantly over time across all body areas (see Table 1 and Figure 2). Pairwise comparisons showed the clinically meaningful discomfort increases from baseline that were apparent by 90 or 120 min were also statistically significant for the low back (120 min IRR = 4.20, *p* ≤ 0.001) and hip/thigh/buttock (90 min: IRR = 14.67; 120 min IRR = 19.75, *p* ≤ 0.001).

**Figure 3:**
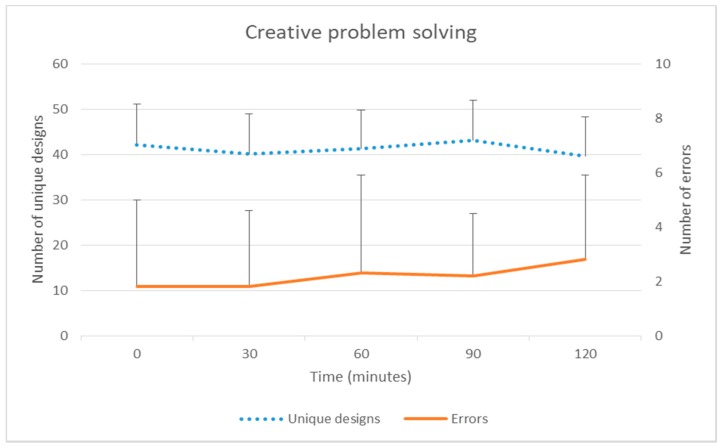
Mean (+standard error) creative problem solving (unique designs and errors) over two hours of prolonged sitting (non transformed).

There was no significant change over time in sustained attention (No-go success or reaction time). While the mean number of creative problem solving unique designs did not change significantly over time, errors increased significantly over time (group mean at baseline 1.8 [SD 3.2] to 2.8 [3.1] at 120 min) with pairwise testing (compared to baseline) also statistically significant at 120 min (IRR 1.05, *p* = 0.036) (Table 2 and Figure 3). Perceived mental state deteriorated over time. Pairwise testing (compared to baseline) showed statistically significant differences at 90 min (β = 7.47, *p* < 0.001) and 120 min (β = 9.28, *p* < 0.001).

Samples were taken approximately every 3 min to ensure consistency of the data. Samples either side of those chosen were visually similar. Based on visual inspection for artefacts and checking outliers, EMG data were excluded for specific time points of one participant’s erector spinae, five participants’ biceps femoris and two participants’ external oblique. Amplitude and median frequency of erector spinae, trapezius, rectus femoris, biceps femoris and external oblique muscles did not change significantly over the two hours (Table 3). Low back angle (sagittal mean) appeared to change from −5.9° (group mean at baseline) [SD 15.6] to −0.5° [13.4] at 120 min into less lordosis and closer to usual sitting posture (group mean sitting posture −5.1°). Pelvis movement appeared to increase from 1.6 cm/s [1.0] at baseline to 2.2 cm/s [1.4] at 120 min over the two hours. However there was no significant time effect for low back angle or pelvis movement.

### Correlations

Low back discomfort was substantially negatively correlated with external oblique median frequency (r = −0.533) but not with external oblique amplitude, or erector spinae (amplitude or median frequency), low back angle (mean or standard deviation) or pelvis movement (see Table 4). Lower limb discomfort was not significantly correlated with biceps femoris and rectus femoris muscle amplitude or median frequency, or pelvis movement (see Table 5). Upper limb discomfort was not correlated with trapezius amplitude or median frequency or pelvis movement (see Table 6). Total body discomfort had a moderate correlation with creative problem solving errors (rho = 0.480, *p =* 0.032), approached significance with mental state (rho = 0.423, *p =* 0.063), however was not significantly correlated with unique designs, No-go success, or reaction time (see Table 7). 

## 4. Discussion

The current study examined discomfort, cognitive function, muscle fatigue, low back angle, pelvis movement and mental state over two hours of prolonged sitting. Discomfort increased significantly across all body areas with low back rated highest. There was a deterioration in creative problem solving errors over time and a negative impact on mental state during prolonged sitting. There were no effects on muscle fatigue, low back angle or pelvis movement over time.

In congruence with a number of laboratory studies, discomfort increased with time for the low back [12], lower limb [18] and also the upper limb [51]. Clinically meaningful increases were evident for low back (10 participants) and hip/thigh/buttock (nine participants) discomfort. Discomfort related to sitting is thus a potentially important issue for office workers, requiring greater understanding and consideration of interventions.

Low back discomfort had a clinically meaningful increase in discomfort at the end of the 120 min of prolonged sitting, suggesting a posture break should be taken before 120 min of prolonged sitting. Despite low back discomfort being correlated with an external oblique fatigue indicator (median frequency), there was no evidence of erector spinae or external oblique fatigue (i.e., increased amplitude or decreased median frequency) over the two hours of sitting. While evidence suggests sitting can result in increased erector spinae muscle activation, muscle activity level varies depending on the posture assumed [20,52]. There was a change in low back angle to less lordosis over time, which is in line with prior evidence [7,53]. Castanharo et al. [54] has previously suggested passive tissue stress to be less with greater anterior tilt and the lumbar spine closer to neutral, resulting in less discomfort. Although not evident in the results from this study, it is postulated that over a longer duration the increase in posterior tilt may contribute to more passive tissue stress and thus discomfort. In contrast, the lack of increase in pelvis movement was not expected. O’Sullivan et al. [55] found those with discomfort adopted a more static end-range sitting position with less frequent micro-movements, but large infrequent shifts in posture during sitting. This is in line with Fenety et al. [56] who found fidgets increased with sitting time. The data capture sampling period of 10 s in the current study may have missed irregular movement and thus not reflected the full amount of movement undertaken. Therefore whilst not evident in our study, the lack of movement may have been a contributor to discomfort. Further research of movement patterns during prolonged sitting preceding discomfort, may help to understand the adoption of preventative movement strategies versus movement to alleviate discomfort.

Hip/thigh/buttock had a clinically meaningful increase in discomfort at 90 min which was also statistically significant (which was earlier than for the low back). Discomfort in the hip/thigh/buttock area is postulated to have some relationship with gluteal pressure [17]. Sondergaard, Olesen, Sondergaard, de Zee and Madeleine [11] separated buttock and thigh regions and found discomfort in the buttock was rated considerably higher than the thigh. This may in part be attributed to the pressure distribution in sitting. Makhsous et al. [20] found a concentration of higher pressure around the ischial region of the buttocks compared to the thigh. In the remainder of the lower limb, although knee and ankle/foot discomfort increased over time neither reached clinically meaningful levels. Winkel and Jorgensen [18] studied eight hours of seated work and found increased foot swelling and decreased foot temperature, when there was minimal leg movement in sitting. Lower limb discomfort was not correlated with pelvis movement in the current study. It was postulated that increased pelvis movement may assist to relieve discomfort in the gluteal region [11] but potentially has less benefit for the lower leg. There may be other factors which were not measured, such as swelling and blood flow, which may help to understand mechanisms underlying lower limb discomfort. Further research which separates thigh and buttock discomfort measures and considers lower limb swelling may help to understand the mechanisms for buttock and lower leg discomfort better.

Despite statistically significant increases in discomfort in all upper limb areas, changes from baseline did not reach clinically meaningful levels. The increase in the neck and shoulder discomfort appeared greater than elbow and wrist/hand increases. Neck discomfort for office workers has been found in a number of studies [21,57] and has been associated with neck flexion. For the upper limb, field and laboratory studies have found discomfort to be greater in just-sitting than other work postures such as sit-stand [23,24]. This finding has been postulated to be a result of increased loading on neck and shoulder muscles when sitting [23]. In the current study participants had autonomy over the tasks undertaken and as a result there may have been individual differences in duration of a specific posture (such as neck flexion) or repetitive movements (e.g., using mouse or keyboard). In the workplace there may be more or less autonomy in task performance and duration which may influence discomfort. As this study was for two hours duration, discomfort may increase more over a longer duration and thus reach clinically meaningful levels. To gain a clear understanding of neck and upper limb discomfort in office workers an accurate description of the pattern of tasks performed may be important.

The current study found a decline in cognitive function over prolonged sitting in the form of increased creative problem solving errors, although performance in generating unique designs did not change over time and there was no change in sustained attention. Mental state was perceived to decline from 90 min. The increase in errors is consistent with other evidence showing working in prolonged positions led to poorer cognitive function than working with interruption and adoption of an alternate work position [58]. On the other hand, some studies have failed to find a significant difference in cognitive function (including executive tasks, memory and attention) over periods of uninterrupted sitting [29] and in studies with shorter periods [59]. It is noted, however, that not all studies of cognitive function have considered the same attributes or over the same length of time which reduces the ability to make direct comparisons [60]. The results of the current and other studies show that prolonged uninterrupted sitting can negatively impact cognitive function.

For sustained attention this study found no significant change in reaction time and No-go success. It is known that sustained attention has a tendency to deteriorate with time-on-task [61]. In the current study, the lack of decrement may have been due to the self directed tasks performed by participants being able to keep participants relatively alert. Alternatively the testing itself (approximately 4 min every 30 min) may have been perceived as a novelty and resulted in an increase in attention. Interestingly although sustained attention was maintained, there was a concurrent finding of deterioration in mental state. However the change in mental state was relatively small given the possible response range. In considering wider measures of attention and perceived mental state, other studies have considered mental fatigue. Wennberg et al. [29] found there was an increase in mental fatigue over four hours of prolonged sitting, along with a decrease in heart rate and altered neuroendocrine biomarkers, potentially reflecting an influence on the autonomic nervous system. It has been suggested that the relatively low energy expenditure and metabolic rate of prolonged sitting [33] has potential to negatively impact brain health [25] and thus potentially effect cognitive function. In contrast, higher energy expenditure has been linked with changes in metabolism including cerebral blood flow [32] and oxygenation [62]. It is clear that the factors influencing cognitive function and mental state are likely to be multifaceted. Research comparing just-sitting to alternate work positions with higher energy expenditure may provide greater understanding.

Results of the current study showed a significant moderate correlation between total body discomfort and cognitive function errors and moderate but not statistically significant association with mental state. Pronk et al. [31] found in a field study that by reducing periods of prolonged sitting there was reduced pain and improved productivity and focus. However, studies which have considered the correlation between discomfort and cognitive function are limited and have focused on integrated work productivity tasks such as typing rather than more discrete cognitive function tests [63,64]. A change in physical state, such as discomfort, has been hypothesised to be related to changes in the allocation of attention resources [65] although this was not conclusively evident through the measures in this study. Whilst the current study found some evidence of acute deterioration in cognitive function during sitting, there are also concerns that chronic sedentary behaviour has potential to negatively influence cognitive function more substantially [25,26]. Research of mechanisms not considered in this study, such as autonomic nervous system activity, may assist in understanding the long term effect of chronic uninterrupted seated work on cognition.

## 5. Strengths and Limitations

This study used a strong design (within participant, repeated measures) and included an elaborate range of variables to characterise the effect of prolonged sitting on discomfort and cognitive function as well as potential mechanisms such as muscle fatigue and lumbar posture and movement. It is acknowledged however that the convenience sample, laboratory setting and test protocol may have influenced the results and thus generalising results should be undertaken with caution. For example the sensors on their low back may have impacted discomfort ratings. In addition data capture may not have been of sufficient duration to show changes for some sporadic or irregular movement. The lightly controlled tasks performed by participants may have increased random variance. One person did stand once (briefly) during the two hours, however their level of discomfort did not influence overall findings. It is also acknowledged that individual factors such as motivation may have influenced cognitive function results. Further it is acknowledged that correlation results outlined which are suggestive of a relationship between variables require further exploration. The number of statistical tests performed raises the issue of type 1 errors, the over interpretation of which was minimised by examining the pattern of response over repeated measures and across multiple dependent variables.

## 6. Conclusions

This study found acute negative effects during two hours of prolonged sitting with clinically meaningful increases in discomfort in the low back and hip/thigh/buttock areas. Regarding cognitive function, some deterioration in creative problem solving was observed, but there was no impact on sustained attention during prolonged sitting. No significant changes in muscle activation, low back angle and pelvis movement were found. Increasing body discomfort had a moderate correlation with cognitive function suggesting potentially important relationship between them. The observed findings suggest sitting for prolonged periods may have consequences for musculoskeletal discomfort and cognitive function in the short term and breaks to change position are recommended.

## Figures and Tables

**Table 1:** Discomfort [mean (standard deviation)] over 2 h of prolonged sitting with incident rate ratio (IRR) for effect of time.

Variable	Minutes—Group Means (SD)					Time Effect		
	0	30	60	90	120	IRR	Conf Interval	*p* Value
**Discomfort (/100)**								
Neck	3.1 (4.2)	3.6 (3.6)	8.7 (10.1)	11.8 (14.9)	11.6 (17.3)	1.38	1.19–1.61	<0.001
Shoulder	2.2 (4.4)	3.1 (3.4)	7.9 (10.9)	10.0 (15.5)	11.1 (17.6)	1.47	1.29–1.67	<0.001
Elbow	0.9 (2.4)	1.9 (2.8)	2.4 (3.9)	3.3 (5.0)	2.4 (3.1)	1.28	1.11–1.47	0.001
Wrist/hand	0.7 (1.6)	1.4 (2.4)	2.4 (4.8)	2.3 (4.3)	2.6 (5.1)	1.30	1.12–1.52	0.001
Upper back	3.5 (7.8)	4.5 (7.6)	8.0 (10.1)	10.8 (15.6)	11.7 (15.4)	1.44	1.25–1.67	<0.001
Low back	4.8 (7.2)	5.5 (6.8)	7.9 (8.4)	12.2 (12.8)	16.3 (14.3) *	1.47	1.32–1.65	<0.001
Hip/thigh/buttock	1.1 (2.7)	2.2 (4.8)	5.8 (8.8)	11.5 (13.8) *	14.8 (17.5) *	2.19	1.81–2.65	<0.001
Knee	1.5 (3.1)	1.7 (3.2)	3.7 (5.9)	4.7 (8.5)	3.8 (6.8)	1.33	1.16–1.53	<0.001
Ankle/foot	1.0 (2.9)	1.5 (2.1)	2.6 (4.4)	2.9 (3.9)	3.7 (5.5)	1.42	1.20–1.70	<0.001
Upper limb	1.3 (2.0)	2.1 (2.0)	4.2 (5.1)	5.2 (7.1)	5.4 (7.0)	1.38	1.27–1.50	<0.001
Lower Limb	1.2 (2.7)	1.8 (2.6)	4.1 (5.2)	6.3 (7.5)	7.4 (7.5)	1.66	1.48–1.86	<0.001
Total body	2.1 (2.8)	2.8 (2.5)	5.5 (5.0)	7.7 (7.0)	8.6 (7.7)	1.43	1.33–1.53	<0.001

Confidence Interval is 95th, * statistically significant pairwise comparisons of clinically meaningful increases from baseline.

**Table 2:** Cognitive function [mean (standard deviation)] over 2 h of prolonged sitting with coefficient (Beta) for effect of time.

Variable	Minutes—Group Means (SD)					Time Effect		
	0	30	60	90	120	Beta	Conf Interval	*p* Value
**Sustained attention**								
no-go success (%)	59.4 (29.7)	57.6 (30.1)	54.8 (30.1)	56.2 (27.5)	54.4 (30.7)	−1.14	−2.68–0.40	0.148
reaction time (msec)	375.9 (73.3)	365.4 (68.1)	361.2 (74.1)	373.1 (66.8)	365.5 (62.6)	−1.30	−5.2–2.81	0.534
**Problem Solving**								
unique designs (n)	42.1 (9.1)	40.2 (8.8)	41.3 (8.5)	43.2 (8.7)	39.6 (8.7)	−0.22	−0.69–0.26	0.372
errors (n)	1.8 (3.2)	1.8 (2.8)	2.3 (3.6)	2.2 (2.3)	2.8 * (3.1)	0.25	0.03–0.47	0.026

Confidence Interval is 95th, * statistically significant pairwise comparisons from baseline.

**Table 3:** Muscle fatigue, low back angle and movement, calf swelling and mental state [mean (standard deviation)] over 2 h of prolonged standing with coefficient (Beta) for effect of time.

Variable	Minutes—Group Means (SD)					Beta	Confidence Interval	*p* Value
	0	30	60	90	120			
**Muscle Fatigue (A—Amplitude (% Reference Contraction), MF—Median Frequency [hertz])**								
erector spinae—A	25.6	24.3	20.8	18.2	18.1	1.05	0.81–1.10	0.532
	(48.3)	(30.3)	(19.0)	(16.0)	(18.3)			
erector spinae—MF	84.4	82.7	87.7	100.0	99.3	1.10	1.00–1.17	0.065
	(40.1)	(38.0)	(44.4)	(49.0)	(55.2)			
trapezius—A	47.6	36.1	46.8	41.2	31.1	0.98	0.81–1.15	0.710
	(124.6)	(86.7)	(113.1)	(112.6)	(49.8)			
trapezius—MF	73.3	71.3	70.2	68.4	72.0	1.00	0.95–1.02	0.459
	(16.2)	(15.5)	(14.1)	(13.2)	(15.5)			
rectus femoris—A	20.2	19.2	23.2	21.2	25.3	0.98	0.89–1.10	0.620
	(36.7)	(36.8)	(38.2)	(52.2)	(48.0)			
rectus femoris—MF	107.4	105.6	99.4	120.3	92.6	0.98	0.85–1.12	0.786
	(68.7)	(72.5)	(67.9)	(81.0)	(51.6)			
biceps femoris—A	10.1	11.3	12.7	9.9	12.5	0.93	0.95–1.29	0.206
	(8.2)	(8.1)	(14.3)	(7.7)	(16.4)			
biceps femoris—MF	164.7	158.9	151.1	186.7	152.0	1.00	0.89–1.15	0.884
	(63.1)	(68.0)	(82.0)	(63.4)	(64.1)			
external oblique—A	17.2	24.9	21.9	22.6	21.5	1.04	0.92–1.20	0.489
	(15.7)	(28.3)	(20.9)	(20.6)	(20.1)			
external oblique—MF	77.4	76.8	63.4	70.6	68.5	0.94	0.87–1.26	0.151
	(37.8)	(50.3)	(31.7)	(38.1)	(39.4)			
**Low back angle (degrees)**								
sagittal mean	−5.9	−2.8	−3.3	−3.7	−0.5	0.98	−0.25–2.21	0.117
	(15.6)	(17.0)	(17.4)	(14.1)	(13.4)			
sagittal std	0.3	0.7	1.0	0.4	0.8	0.09	−0.04–0.23	0.172
deviation	(0.3)	(0.7)	(1.5)	(0.4)	(1.4)			
**Pelvis movement (cm/s)**								
distance	1.6	1.9	2.3	1.9	2.2	1.11	0.95–1.3	0.178
	(1.0)	(0.8)	(1.9)	(1.0)	(1.4)			
**Mental state (/100)**								
perceived mental state	28.0 (18.8)	32.4 (19.3)	31.1 (16.4)	35.4 * (19.6)	37.2 * (19.1)	2.16	1.10–3.22	<0.001

Confidence Interval is 95% confidence interval, ^ back transformed, * statistically significant pairwise comparisons from baseline.

**Table 4:** Change score correlations (r) for low back discomfort and low back angle, pelvis movement and muscle fatigue amplitude (A) and median frequency (MF) measures over 2 h prolonged sitting.

	Low Back Discomfort	Usual Sit (Mean Sagittal)	Usual Sit (SD Sagittal)	Erector Spinae (A)	Erector Spinae (MF)	External Oblique (A)	External Oblique (MF)	Pelvis Movement
**Low Back Discomfort, r**	1.000							
**Usual Sit (Mean Sagittal), r**	−0.269	1.000						
**(*p* Value)**	0.252							
**Usual Sit (SD Sagittal), r**	0.297	−0.422	1.000					
**(*p* Value)**	0.204	0.064						
**Erector Spinae (A), r**	−0.140	−0.290	0.477	1.000				
**(*p* Value)**	0.569	0.229	0.039					
**Erector Spinae (MF), r**	0.374	−0.175	−0.263	−0.489	1.000			
**(*p* Value)**	0.115	0.474	0.277	0.034				
**External Oblique (A), r**	0.170	0.153	0.058	0.036	−0.117	1.000		
**(*p* Value)**	0.530	0.571	0.831	0.894	0.665			
**External Oblique (MF), r**	−0.533	0.427	−0.123	0.058	−0.461	−0.175	1.000	
**(*p* Value)**	0.028	0.087	0.638	0.824	0.062	0.516		
**Pelvis Movement, r**	0.380	−0.310	0.760	0.582	−0.348	0.079	−0.014	1.000
**(*p* Value)**	0.098	0.184	< 0.001	0.009	0.144	0.772	0.959	

**Table 5:** Change score correlations (r) between lower limb discomfort, muscle fatigue [amplitude (A) and median frequency (MF)] and pelvis movement over 2 h prolonged sitting.

	Lower Limb Discomfort	Biceps Femoris (A)	Biceps Femoris (MF)	Rectus Femoris (A)	Rectus Femoris (MF)	Pelvis Movement
**Lower Limb Discomfort, r**	1.000					
**Biceps Femoris (A), r**	−0.114	1.000				
**(*p* Value)**	0.652					
**Biceps Femoris (MF), r**	0.072	−0.510	1.000			
**(*p* Value)**	0.799	0.052				
**Rectus Femoris (A), r**	0.288	0.118	0.221	1.000		
**(*p* Value)**	0.233	0.653	0.447			
**Rectus Femoris (MF), r**	−0.084	−0.291	0.312	−0.595	1.000	
**(*p* Value)**	0.734	0.258	0.277	0.007		
**Pelvis Movement, r**	0.243	0.729	−0.361	0.310	−0.196	1.000
**(*p* Value)**	0.301	0.001	0.186	0.196	0.421	

**Table 6:** Change score correlations (r) between upper limb discomfort, muscle fatigue [amplitude (A) and median frequency (MF)] and pelvis movement over 2 h prolonged sitting.

	Upper Limb Discomfort	Trapezius (A)	Trapezius (MF)	Pelvis Movement
**Upper Limb Discomfort, r**	1.000			
**Trapezius (A), r**	0.101	1.000		
**(*p* Value)**	0.673			
**Trapezius (MF), r**	0.102	0.501	1.000	
**(*p* Value)**	0.668	0.022		
**Pelvis Movement, r**	−0.168	0.234	0.558	1.000
**(*p* Value)**	0.479	0.281	0.011	

**Table 7:** Change score correlations (rho) between total body discomfort, creative problem solving, sustained attention and mental state over 2 h prolonged sitting.

	Total Body Discomfort	Creative Problem Solving		Sustained Attention		Mental State
		Unique Designs	Errors	No-Go Success	Reaction Time	
**Total body Discomfort, rho**	1.000					
**Unique Designs, rho**	0.157	1.000				
**(*p* Value)**	0.508					
**Errors, rho**	0.480	−0.294	1.000			
**(*p* Value)**	0.032	0.208				
**No-go Success, rho**	−0.121	0.292	0.200	1.000		
**(*p* Value)**	0.611	0.212	0.397			
**Reaction Time, rho**	−0.053	0.383	0.101	0.795	1.000	
**(*p* Value)**	0.823	0.096	0.672	<0.001		
**Mental State, rho**	0.423	−0.226	0.398	0.013	−0.028	1.000
**(*p* Value)**	0.063	0.338	0.082	0.957	0.906	
